# Development of QSAR Models and Web Applications for Predicting hDHFR Inhibitor Bioactivity Using Machine Learning

**DOI:** 10.3390/molecules30234618

**Published:** 2025-12-01

**Authors:** Ibrahim Maattallaoui, Mahamadou Sakho, Abdellah Maatallaoui, Enrique Barrajón-Catalán, Noureddine El Aouad

**Affiliations:** 1 Laboratory of Life and Health Sciences, Faculty of Medicine and Pharmacy of Tangier, Abdelmalek Essaadi University, Road of Rabat 15 km Gzenaya BP 365 Tanger, Tetouan 92000, Morocco; ibrahim.maattallaoui@etu.uae.ac.ma (I.M.); mahamadou.sakho@etu.uae.ac.ma (M.S.); 2 Laboratory of Advanced Science and Technologies, Polydisciplinary Faculty—Larache (FPL), Abdelmalek Essaadi University, Tetouan 92000, Morocco; abdellah.maatallaoui@etu.uae.ac.ma; 3 Institute of Research, Development and Innovation in Health Biotechnology of Elche (IDiBE), Universitas Miguel Hernández (UMH), 03202 Elche, Spain

**Keywords:** hDHFR, ML-QSAR, random forest algorithm, machine learning, bioactivity prediction

## Abstract

Human dihydrofolate reductase (hDHFR) is a crucial cellular enzyme in folate metabolic pathway, where it catalyzes the reduction of dihydrofolate into tetrahydrofolate (THF) and an important cofactor involved in DNA, RNA, protein biosynthesis and cell proliferation. Due to its importance, hDHFR has become a promising target for therapeutic development, particularly in treating cancer, bacterial infections, and autoimmune diseases. Its inhibition has found clinical value in antitumor, antimicrobial and antiprotozoal treatment; however, the emergence of resistance to existing hDHFR inhibitors necessitates the development of new and more potent compounds. In the current study, we propose a cheminformatics-based approach using machine learning to develop predictive models of hDHFR bioactivity. We used three types of molecular descriptors in the form of fingerprints, i.e., PubChem, Substructure, and MACCS, to capture structural properties associated with hDHFR inhibition. Predictive models were built using a random forest algorithm optimized through hyperparameter tuning. Feature selection was performed using Recursive Feature Elimination (RFE), and dataset dimensionality was reduced by removing outliers through Principal Component Analysis (PCA) to optimize model performance and reducing overfitting and weak predictivity. The resulting models are validated through external test sets, domain applicability analysis, and interpretation of influential molecular features via random forest feature importance selection plots and correlation matrix analysis. All three models exhibited strong predictive capabilities, with R-squared (R^2^) values ranging from 0.9849 to 0.9934 for the training set and 0.9381 to 0.9591 for the test set. These final predictive models were further incorporated into an accessible web application, enabling users to estimate the bioactivity of new compounds targeting hDHFR.

## 1. Introduction

Enzymes are proteins able to catalyze essential biochemical reactions [1]. In the therapeutic field, the selective inhibition of enzymes by drug treatment allows them to block or limit some of their biochemical reactions. These biochemical reactions may be directly or indirectly linked to the overexpression of certain diseases. In medicinal chemistry, dihydrofolate reductase (DHFR) has gained attention as a promising therapeutic target, especially in the treatment of conditions such as cancer, bacterial infections, malaria and tuberculosis [2,3,4,5,6]. This is because many cancer and bacterial cells need folate to grow and maintain rapid proliferation [7]. Folic acid, a water-soluble vitamin, is biologically inactive in its original form, must be converted to tetrahydrofolate (THF), a metabolically active cofactor, which is required for the synthesis of purines, thymidylate, and certain amino acids [8]. That is why, an absence of tetrahydrofolate is correlated with an inhibition of cell division and growth [9]. Furthermore, hDHFR plays a crucial role, facilitating the conversion of dihydrofolate to tetrahydrofolate using NADPH as a coenzyme, and consequently participating in the synthesis of the substrate involved in cell proliferation [8]. Tetrahydrofolate is synthesized and converted into 5′,10′-methylenetetrahydrofolate, which is used by thymidylate synthetase to catalyze the first stage of DNA synthesis, then, a precursor of DNA synthesis, deoxyuridine monophosphate is converted to deoxythymidine, accompanied by the oxidation of 5,10-methylenetetrahydrofolate to dihydrofolate [10]. Thus, selective inhibition of DHFR reduces the quantity of tetrahydrofolates produced for pyrimidine and purine synthesis. As a result, cancer cells die because RNA and DNA synthesis are blocked [10]. This inhibition also leads to bacterial death [7]. Nowadays, several hDHFR inhibitors used to treat cancer and bacterial infections have been approved by the FDA and/or EMA, including methotrexate (lymphoma, leukemia), raltitrexed (colorectal cancer), pemetrexed (treat peripheral T-cell lymphoma), Pyrimethamine (infections caused by parasites such as malaria), and trimethoprim (urinary tract infections) [10,11,12].

Given the importance of this enzyme in therapeutic treatment, and the increasing resistance to treatment agents including anticancer, antibacterial, antitubercular, and antifungal drugs [13], there is an urgent need to discover more effective molecules to inhibit hDHFR. AI-driven approaches, including machine and deep learning, have transformed drug discovery by improving prediction accuracy while reducing development time and costs using drugs known safety profiles [14]. These include ADMET prediction, molecular docking, QSAR, pharmacophore modeling, MD simulations, and DFT studies, which contributes substantially in drug discovery and development [15,16,17,18,19,20,21]. In the search for new active therapeutic molecules, or to improve the efficacy of existing bioactive molecules, these techniques are increasingly used in combination combined with in vitro and in vivo tests [22,23,24]. Machine learning (ML) methods are increasingly applied in QSAR studies because they able to identify the relationships between chemical structure and biological activity, efficiently select relevant features, and improve both the accuracy and predictive performance of the model [25,26]. Quantitative structure–activity relationship (QSAR) modeling studies play a central role for predicting the biological activity of molecules, when experimental data or resources are limited [27], so it has become one of the effective prediction methods of molecular properties early in drug discovery. This is a powerful technique that constructs mathematical models correlating compound’s bioactivity with its structural characteristics, physicochemical properties, and other descriptors [28]. It can assess how different functional groups may contribute to certain biological activities, helping to identify which parts of a molecule are responsible for its effects [29]. A QSAR model analyzes how the chemical structure of a compound relate to its target properties, using either linear or nonlinear approaches, aiming to reveal patterns within complex and high dimensional datasets [30]. In QSAR modeling, machine learning algorithms are used to improve model accuracy and prediction capabilities, based on large datasets containing chemical compounds and their biological activity, making them valuable tools for discovering effective compounds capable of effectively interacting with therapeutic targets in disease treatment [31]. To develop QSAR models, researchers apply different techniques to generate input features and utilize computational methods based on machine learning algorithms, including Multiple Linear Regression (MLR), Partial Least Squares (PLS), Decision Trees (DT), Random Forests (RF), Support Vector Machines (SVM) [32,33,34,35]. These algorithms learn from compounds with already known activity and utilize their structural and physicochemical features to predict the activity of new compounds [28].

This study aimed to establish a computational pipeline for constructing three distinct ML-QSAR models, each based on different molecular descriptors, to predict the inhibitory potential of compounds targeting hDHFR enzyme. The approach uses PubChem, Substructure and MACCS fingerprints, along with feature selection and dimensionality reduction techniques like recursive feature elimination (RFE) and principal component analysis (PCA). The selected predictive models are integrated into a Python-based web tool (https://hdhfr-prediction.streamlit.app/, accessed on 10 September 2025) to predict pIC_50_ exhibited by small molecules.

## 2. Results and Discussion

### 2.1. Data Collection and Preparation

Bioactive molecules that inhibit hDHFR enzyme (Target ID CHEMBL202) were retrieved from the CHEMBL database, including their molecular structures in SMILES format and IC50 values in nanomolar (nM). The initial dataset consisted of 1384 compounds. Next, entries with missing values and those with incomplete or absent bioactivity data were both excluded, generating a final dataset containing 1016 compounds that were then used for further analysis, including data pre-processing. To achieve a uniform distributed set of IC_50_ values, these values were converted to their negative logarithmic form (pIC_50_), calculated as −log_10_(IC_50_). The resulting 1016 compounds were then used for further analysis, including data pre-processing.

### 2.2. Exploratory Data Analysis

Lipinski’s rule of five parameters was calculated for all compounds using RDKit software version 2020.03.3.0, including molecular weight (MW), Log *p*, hydrogen bond acceptors (HBA), and hydrogen bond donors (HBD), for all 1016 compounds. Then, Matplotlib version 3.2.2 and Seaborn packages version 0.11.2 were used for graphical analysis to investigate the relationship between these Lipinski descriptors and bioactivity (pIC_50_) values.

Bar plots illustrate that the number of active molecules targeting hDHFR in our dataset is significantly higher than inactive compounds. Both classes show pIC_50_ values between 6.0 and 8.8. Log *p* values for active molecules vary from 0.9 to 3.0 and for inactive molecules range from 0.9 to 3.2. MW of active compounds varies between 330 and 440 Da, whereas the MW of inactive compounds varies between 310 and 430 Da. HBA values in active and inactive molecules are 6 to 8 and 5 to 8, respectively, and HBD values are 2 to 5 for active molecules and 2 to 3 for inactive compounds.

**Figure 1:**
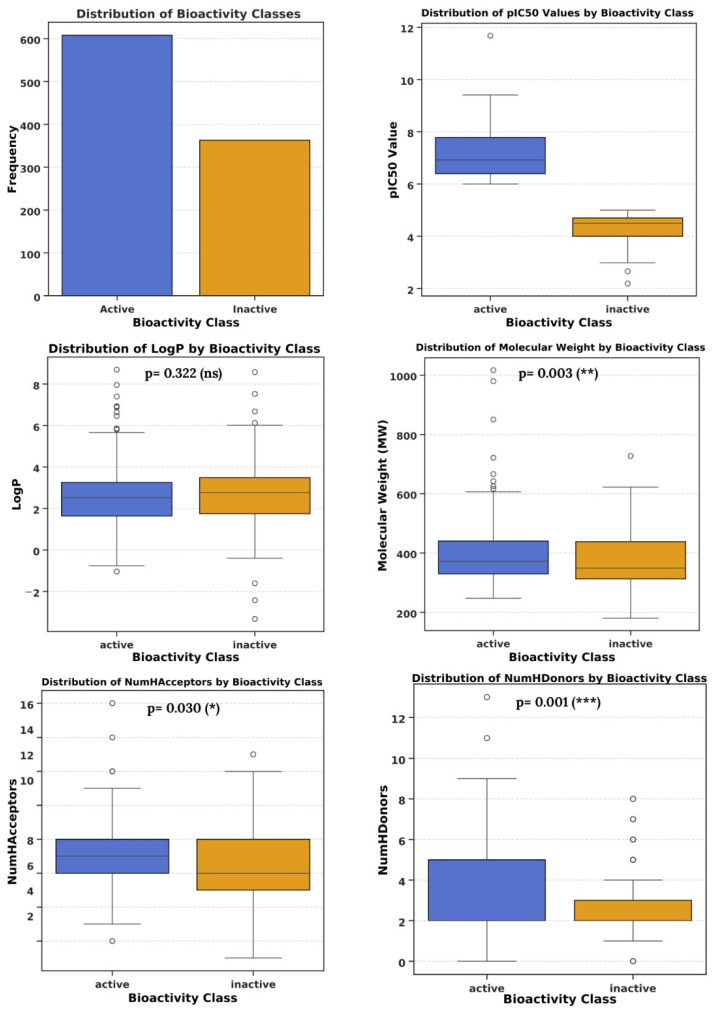
Exploratory data analysis of the curated hDHFR inhibitor dataset. *** *p* < 0.001 (Extremely significant); ** *p* < 0.01 (Very significant); * *p* < 0.05 (Significant); ns *p* ≥ 0.05 (Not significant).

Statistical comparison between active and inactive molecules is summarized in Table 1. Active compounds show slightly higher MW (391.24 ± 91.17 Da) compared to inactive compounds (371.84 ± 86.78 Da; *t* = 2.966, *p* = 0.003, **). HBA is also slightly higher in active molecules (6.98 ± 1.77) than inactive ones (6.69 ± 1.91; *t* = 2.176, *p* = 0.030, *), and HBD is significantly higher in active compounds (3.10 ± 1.72) than in inactive compounds (2.72 ± 1.27; *t* = 3.393, *p* = 0.001, **). In contrast, Log *p* does not differ significantly between active (2.53 ± 1.40) and inactive molecules (2.63 ± 1.40; *t* = −0.991, *p* = 0.322, ns). These results indicate that active compounds tend to have slightly higher molecular weight and hydrogen bonding capacity, which may contribute to their bioactivity, while lipophilicity is not a distinguishing factor. All parameters evaluated in our dataset are consistent with Lipinski’s rule of five, suggesting that the active compounds exhibit favorable drug-like properties (Figure 1).

### 2.3. Molecular Feature Exploration

We utilized the PaDELPy (version 0.1.12) Python wrapper for the PaDEL software version 2.21 to generate three distinct types of molecular fingerprints for our 1016 hDHFR compounds. Specifically, PubChem, Substructure and MACCS fingerprints were computed for each molecule, resulting in separate datasets corresponding to each fingerprint type. Each dataset included the respective pIC_50_ values and SMILES for all compounds. Recursive Feature Elimination (RFE) was then applied to the datasets to identify the 50 most important features from the 881 molecular PubChem features, 308 substructure features, and 166 MACCS features initially calculated.

### 2.4. ML-QSAR Model Optimization

**Figure 2:**
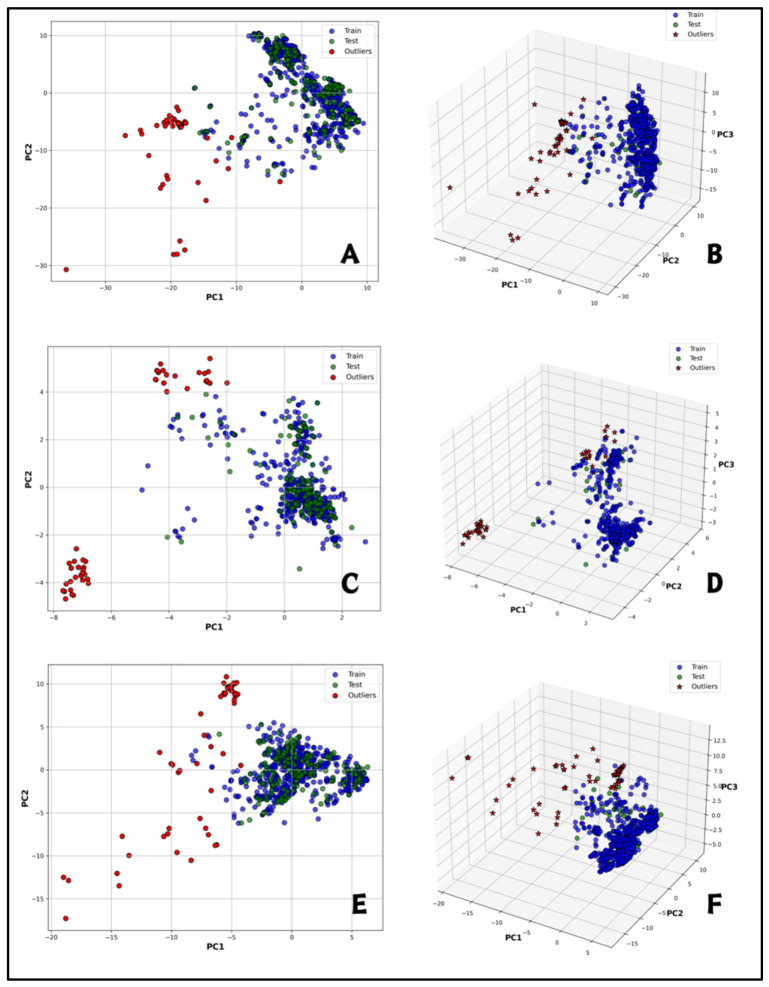
Applicability domain analysis for generated ML-QSAR models, training (blue), test (green) and outliers (red). (**A**,**B**) show 2D and 3D PCA of the PubChem FP prediction model; (**C**,**D**) show 2D and 3D PCA of the substructure FP prediction model; (**E**,**F**) show 2D and 3D analyses of MACCS FP prediction model.

To enhance predictive accuracy at this stage of the study, random forest regression was employed to build more accurate prediction models for the target at this stage of the study. The dataset was divided into 80% for training and 20% for testing, with each model trained using the top 50 selected features. To increase the reliability of our approach we performed an applicability domain (AD) analysis to identify and exclude outliers from each fingerprint-based model (Figure 2). Principal component analysis (PCA) was used to eliminate 49 molecules from PubChem fingerprint model 21 from the substructure fingerprint model and 66 from the MACCS fingerprint model. Hyperparameter tuning was performed to optimize performance for each model based on fingerprints, after outlier removal. Some key parameters were adjusted such as the number of trees (n_estimators), maximum tree depth (max_depth), and the number of features considered for each split (max_features). The best configuration settings were max_depth of 10, max_features set to “sqrt,” and 500 trees for the PubChem model. For the substructure model, a max_depth of 20, max_features set to “log2,” and 500 trees were used. The MACCS model performed best with a max_depth of 50, max_features set to “log2,” and 1000 trees. All these optimizations led to much better accuracy and reliability of predictions across all models. Our optimized random forest model computed using PubChem fingerprints showed good performance metrics, with R^2^ values of 0.9934 for the training set and 0.9591 for the test set. The model also showed low errors with RMSEs of 0.0837 (training) and 0.1848 (testing), MAEs of 0.0593 and 0.1250, MSEs of 0.0070, and 0.0342. These results were based on 774 training molecules and 193 test molecules. In a similar way, the optimized random forest model based on Substructure fingerprints also obtained high R^2^ values of 0.9849 (training) and 0.9381 (testing). RMSE values were 0.1261 for training and 0.2199 for testing, and MAEs of 0.0865 and 0.1381, and MSEs of 0.0159 and 0.0484. This was derived from 796 training molecules and a test set of 199 molecules. Finally, the optimized random forest model using MACCS fingerprints demonstrated R^2^ values of 0.9924 and 0.9381 for the training and test sets, respectively. RMSEs were 0.0919 (training) and 0.2111 (testing), MAEs were 0.0642 and 0.1397, and MSEs were 0.0085 and 0.0446. These performances were based on 769 molecules of training and 181 of testing (Table 2).

**Figure 3:**
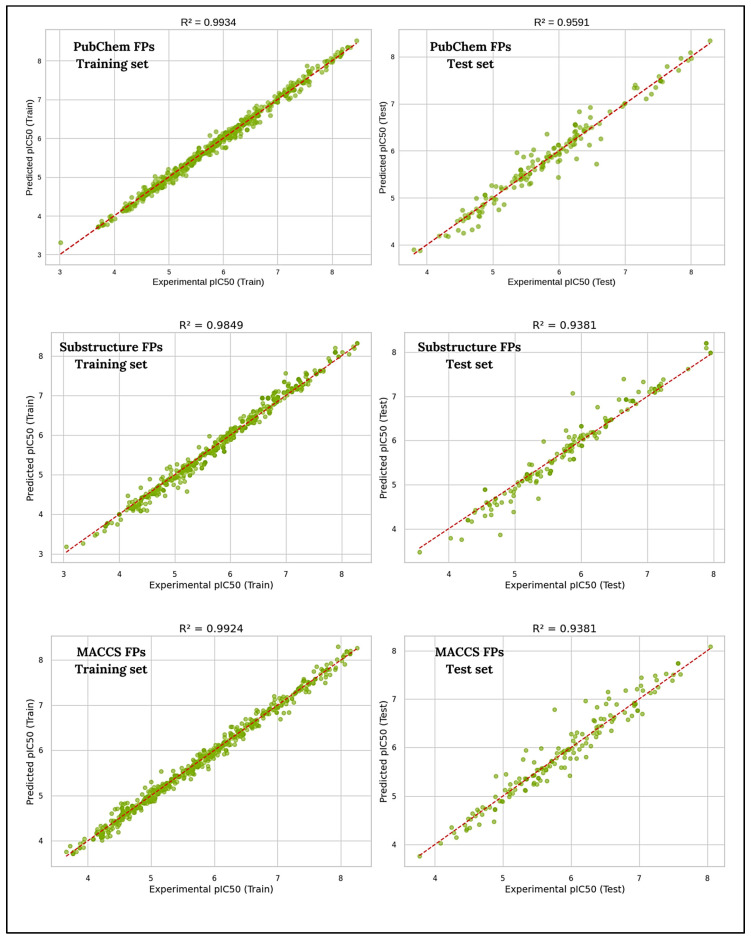
Scatter plot representations of experimental vs. predicted values for all ML-QSAR prediction models.

The robustness of the QSAR models was demonstrated by the established high correlation coefficients of both the training and test datasets, suggesting strong reliability. Scatter plots were generated to provide a visual representation of the models’ performance and to evaluate the predictive capabilities of all models. Regression (scatter) plots for all the generated ML-QSAR models reporting the correlation between pIC_50_ experimentally obtained values (*x*-axis) and the predicted ones (*y*-axis). The plots reveal how well the models performed across both the training and testing sets, with any discrepancies between observed and predicted pIC50 values following a clear pattern, suggesting that the models made reasonable predictions (Figure 3).

**Figure 4:**
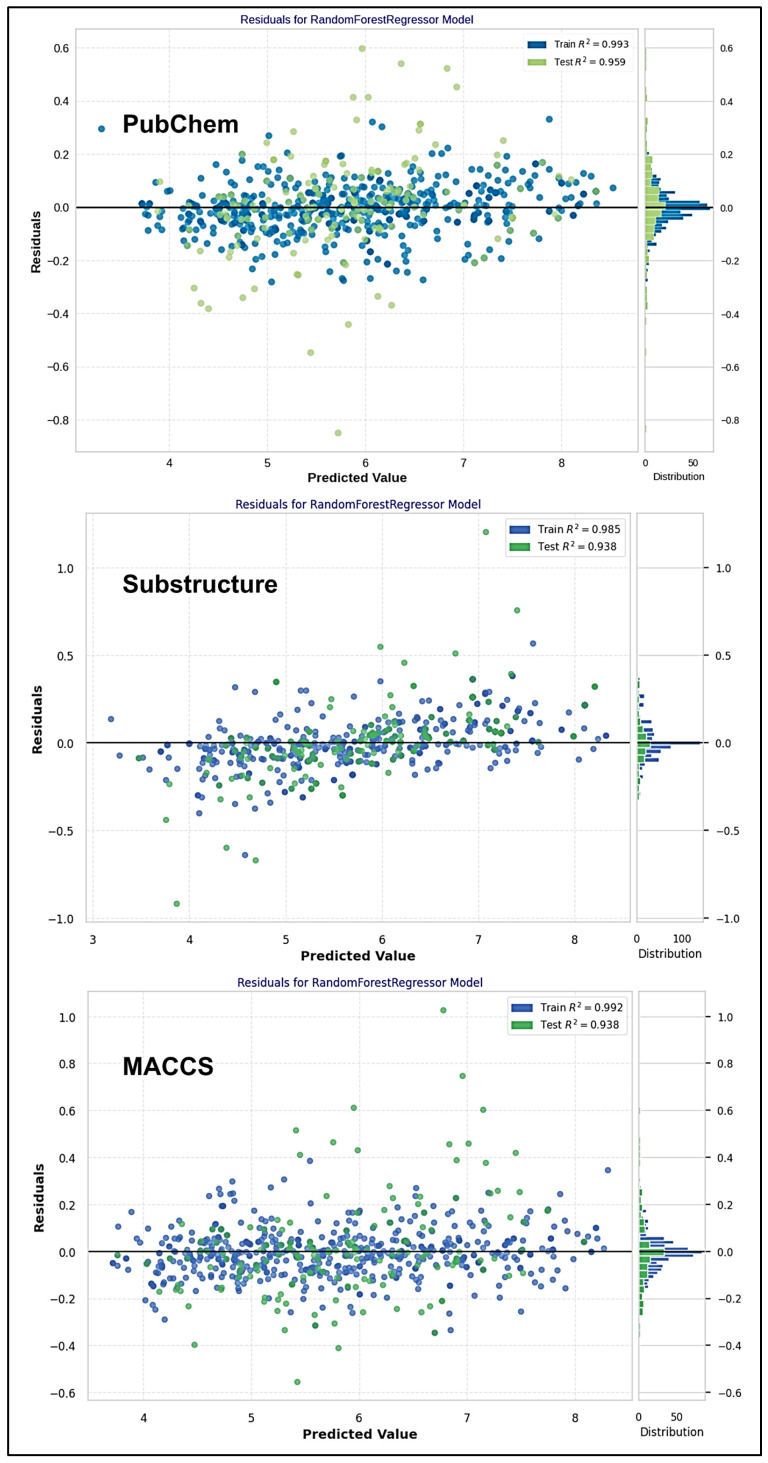
Residual plots for ML-QSAR Models.

The residual plots were used to assess the predictive quality of the random forest regressor (RFR) models, as illustrated in Figure 4. The plots display residuals (*y*-axis) against the predicted values (*x*-axis) of all QSAR models, providing a visual representation of predictive errors across different ranges of predictive values [36]. The plots consist of a set of columns which display residuals from both the training and test datasets, where the training set is displayed in blue color, and the test set is displayed in green. The R^2^ values shown in the plot provide a quantitative assessment of the model performance, with higher values indicating better fit and more accurate predictions.

When reviewing the residuals, it can be observed that the errors are distributed evenly around zero, so there does not seem to be an underlying pattern for over- or under-prediction. Homogeneity also suggests that constant variance for the model’s errors is a favorable characteristic, as a uniform distribution of residuals is observed across predicted values.

This indicates that the model’s predictions are, overall, reliable, with no evident systematic trends in their increasing or decreasing predictions, as the predicted values vary. While there are some outliers with larger residuals, these are infrequent. In general, the model performs quite well, retaining consistent error distribution (homoscedasticity) and consistent predictions. Histograms on the right of the plot show a more detailed view of the residual distribution on the training and test sets. The overall pattern indicates that most residuals are clustered near zero, showing that the model is not significantly over- or under-predicting.

### 2.5. Interpretation of ML-QSAR Models

**Figure 5:**
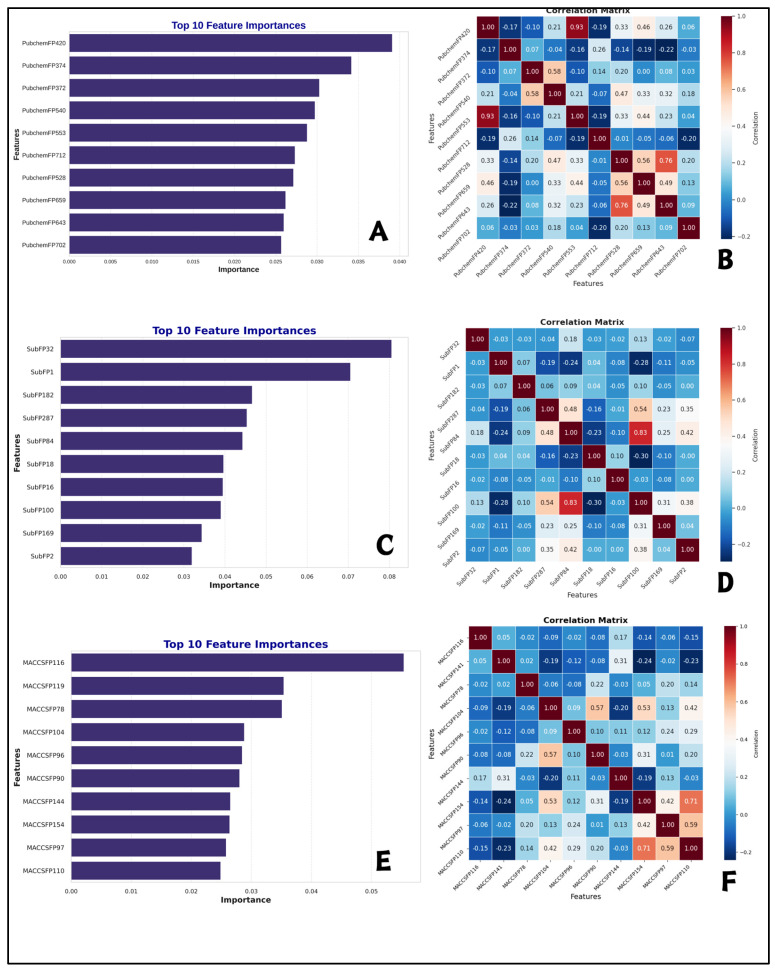
Random Forest Feature Importance (RF-FI) scores and Correlation matrix for the top ten predictive features of each generated ML-QSAR model. (**A**,**B**) PubChem FP prediction model; (**C**,**D**) substructure FP prediction model; (**E**,**F**) MACCS FP prediction model.

Using random forest feature importance in projection analysis, we identified the top ten most significant molecular descriptors among ML-QSAR models (Figure 5). The best features selected were PubchemFP420, PubchemFP374, PubchemFP372, PubchemFP540, PubchemFP553, PubchemFP712, PubchemFP528, PubchemFP659, PubchemFP643, and PubchemFP702 for PubChem prediction model. In the case of Substructure random forest model, we determined the ten most constructive molecular features such as SubFP32, SubFP1, SubFP182, SubFP287, SubFP84, SubFP18, SubFP16, SubFP100, SubFP169 and SubFP2. Finally, the ten best features obtained with MACCS random forest model were MACCSFP116, MACCSFP119, MACCSFP78, MACCSFP104, MACCSFP96, MACCSFP90, MACCSFP144, MACCSFP154, MACCSFP97, and MACCSFP110. These results are detailed in Table 3, which provides a comprehensive summary of the top 10 features identified for each model.

To contextualize these descriptors, we referred to literature docking studies of methotrexate (MTX) in human DHFR (PDB: 1U72), where MTX forms key hydrogen bonds with Ile7, Gln35, Asn64, Arg70, Val115, and Tyr121, as well as carbon–hydrogen, p–alkyl, hydrophobic, and van der Waals interactions with residues including NADPH, Arg32, Ser59, Ile60, Phe31, Trp24, Tyr33, Leu22, Ala9, and surrounding pocket residues [37]. Mapping our top selected features to these interactions provides mechanistic insight. For the PubChemFP model, features such as FP420, FP374, and FP372 likely correspond to aromatic or heterocyclic moieties mimicking the pteridine core of MTX, which participates in key hydrogen bonds. In the Substructure RF model, descriptors like SubFP32 and SubFP1 capture pteridine-like rings, while SubFP287 and SubFP84 reflect groups analogous to MTX’s p-aminobenzoate and glutamate moieties. For the MACCS RF model, features including MACCSFP116 and MACCSFP119 represent aromatic cores contributing to hydrogen bonding and π-alkyl interactions, while MACCSFP104 and MACCSFP96 highlight polar groups involved in the hydrogen-bonding network. Overall, these mappings suggest our models identify chemical features that recapitulate the critical hydrogen bonding, hydrophobic, and van der Waals interactions observed in the MTX-hDHFR complex, thereby lending mechanistic credibility to our predictive frameworks.

Correlation analysis of the selected features revealed a combination of strong, moderate, and weak correlations, each contributing differently to model performance. In the PubChem fingerprints, FP420 and FP540 show a very strong correlation (r = 0.93), and in the Substructure fingerprints, FP100 and FP84 are strongly correlated (r = 0.83). In the MACCS fingerprints, FP144 and FP110 are also highly correlated (r = 0.71). These strong correlations indicate some redundancy, confirming that these features capture similar structural information [38]. At the same time, most other features exhibit moderate and negative correlations, suggesting they provide complementary and independent information, which is advantageous for random forest modeling as it improves diversity and robustness [39]. Overall, these results demonstrate that hDHFR inhibition is influenced by multiple interacting molecular descriptors, collectively contributing to the high predictive performance of the models (Figure 5).

**Figure 6:**
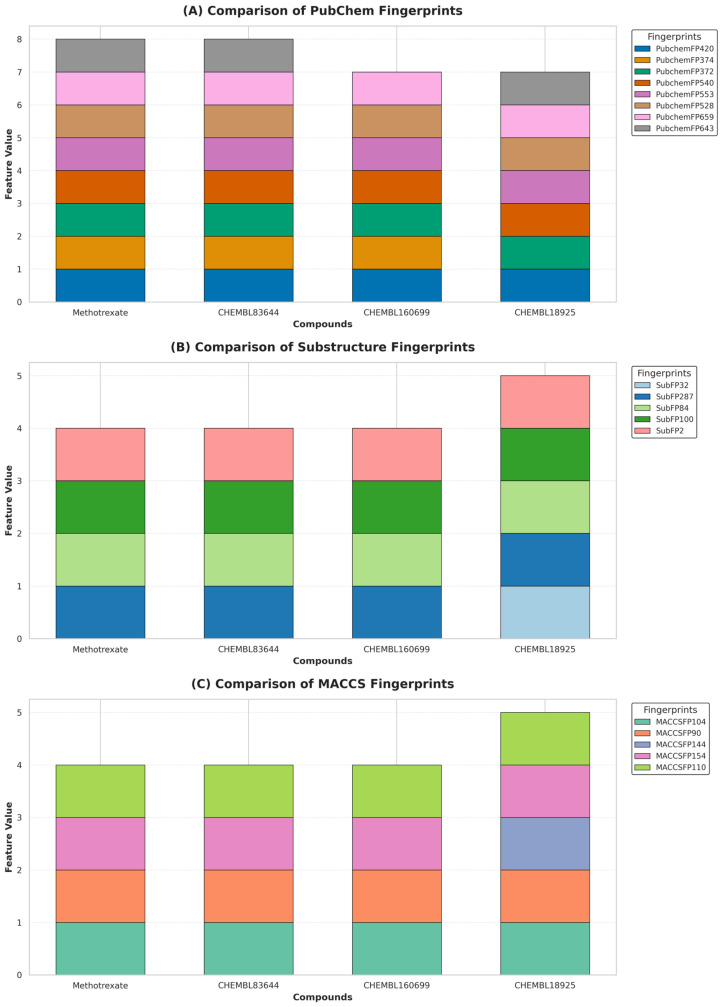
Structural analysis of most active compounds in our dataset compared with approved drug methotrexate. (**A**) ML-QSAR model based on PubChem fingerprints; (**B**) substructure fingerprint model; (**C**) MACCS fingerprint model.

We also conducted a detailed structural analysis of the most active compounds in our dataset, and the FDA-approved hDHFR inhibitor Methotrexate (Figure 6), using a strategy previously reported in [40]. Three compounds CHEMBL83644, CHEMBL160699, and CHEMBL18925 were selected based on their high experimental pIC_50_ values of 8.88, 8.87, and 7.72, respectively, were compared against Methotrexate, which exhibited an experimental pIC_50_ of 9.08. Using the PubChem prediction model combined with random forest algorithm, the predicted pIC_50_ values for CHEMBL83644, CHEMBL160699, CHEMBL18925 and Methotrexate were 8.32, 7.14, 7.36 and 7.65, respectively. All four molecules were subjected to a structural analysis which showed that many of the important PubChem fingerprints were present, for example, both CHEMBL83644 and Methotrexate had PubChemFP420, PubChemFP374, PubChemFP372, PubChemFP540, PubChemFP553, PubChemFP528, PubChemFP659 and PubChemFP64312. CHEMBL160699 possessed all these fingerprints except PubChemFP643, and CHEMBL18925 was absent only of PubChemFP374. These six fingerprints present in all compounds seemed to have the most influence on the high inhibitory activity, suggesting they play a key role in the effectiveness of hDHFR inhibitors. Using the random forest substructure prediction model, it was found that CHEMBL83644, CHEMBL160699, CHEMBL18925 and Methotrexate provided pIC_50_ values of 7.09, 7.09, 7.64, and 7.17, respectively. Methotrexate, CHEMBL83644 and CHEMBL160699 demonstrated the presence of SubFP287, SubFP84, SubFP100, and SubFP2, while CHEMBL18925 contained all these features, plus SubFP32. These five features were identified as the most significant substructure fingerprints amongst the 10 best using VIP plot analysis, suggesting their importance in providing high inhibition of hDHFR. Regarding the random forest based MACCS prediction model predicted pIC50 values were 7.38, 8.26, 7.50, and 7.37 for CHEMBL83644 CHEMBL160699, CHEMBL18925 and Methotrexate, respectively. Methotrexate, CHEMBL83644 and CHEMBL160699 exhibited the presence of MACCSFP104, MACCSFP90, MACCSFP154, and MACCSFP110, whereas CHEMBL18925 contained all these features, plus MACCSFP144. These five features were identified as the most significant substructure fingerprints amongst the 10 best using RF-IF plot analysis, suggesting their importance in providing high inhibition of hDHFR.

A comparison between predicted and experimental pIC_50_ values for the most active inhibitors, including methotrexate, is presented in Table 4. An important limitation of the present models is that they tend to systematically underpredict the bioactivity of the most potent compounds. For instance, methotrexate exhibits an experimental pIC_50_ value of 9.08 in purified enzyme assays, which reflects its maximal intrinsic affinity. In contrast, the models produced lower predicted values of 7.65 (PubChem), 7.17 (Substructure), and 7.37 (MACCS). Although these predictions are closer to the median of the curated ChEMBL dataset (pIC_50_ ≈ 7.66), they do not reproduce the peak potency of this compound.

This trend is characteristic of the high-activity region of the dataset and illustrates a common limitation in QSAR modeling, namely decreased accuracy when extrapolating from sparsely represented chemical space. Machine learning approaches, including Random Forest, often regress extreme values toward the mean when training data are unevenly distributed or when compounds lie outside the densest region of the chemical feature space [41]. Accordingly, predictions for compounds with experimental pIC_50_ values above approximately 8.5 should be interpreted as conservative estimates rather than precise measurements of activity.

This limitation also informs future methodological developments. To improve the sensitivity of the models to ultra-potent compounds, several directions will be pursued: (i) testing additional learning algorithms such as gradient boosting and deep neural networks; (ii) enriching the descriptor space with 3D, quantum-chemical, and interaction-based descriptors; and (iii) employing more refined applicability domain and feature-selection strategies. These improvements are expected to enhance the predictive resolution of the models for high-affinity inhibitors.

### 2.6. Web Application Deployment

A python-based (version 3.7.6) web application named “hDHFR: Bioactivity Prediction App” was developed and made publicly accessible via https://hDHFR-prediction.streamlit.app/, accessed on 10 September 2025. We deployed our work using Streamlit Share platform (https://streamlit.io/, accessed on 5 September 2025), a tool widely used for building and sharing interactive web applications for data science projects [42]. To develop this application, several Python libraries were used like scikit-learn (version 0.23.1), pandas (version 2.2.2), pickles (Python version 3.7.6) as well as standards modules such as subprocesses, os and base64. Input should be compatible with SMILES IDs. After uploading molecular data, the app predicts the inhibitory activity (pIC_50_) of the compounds against hDHFR. The prediction pipeline integrates PaDEL-Descriptor software version 2.21 to calculate molecular fingerprints and then outputs the predicted pIC_50_ values of the provided compounds, to facilitate use for researchers in medicinal chemistry and computational drug design.

## 3. Materials and Methods

**Figure 7:**
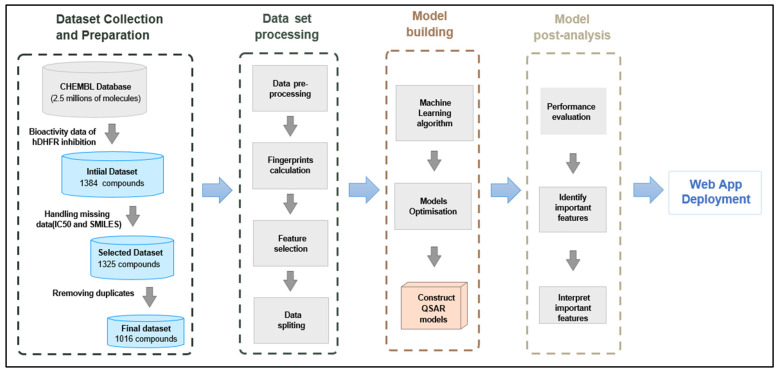
Workflow for ML-QSAR modeling of hDHFR inhibitors.

Figure 7 presents an overview of the workflow used in this study. In brief, QSAR-based models were created to predict and analyze bioactive compounds with inhibitory activity against the human dihydrofolate reductase (hDHFR) enzyme. The study adhered to guidelines of the (OECD) Organization for Economic Cooperation and Development guidelines, which has advertised the main principles to be applied during the development of reliable QSAR models [43]. These guidelines include: (i) a well-defined and clear endpoint; (ii) a transparent unambiguous supervised machine learning method; (iii) a clearly described applicability domain (AD); (iv) appropriate performance metrices to assess accuracy and robustness; and (v) a mechanistic interpretation, if possible.

### 3.1. Data Collection and Preprocessing

Bioactive compounds targeting hDHFR enzyme (ID CHEMBL202) were extracted from CHEMBL database (https://www.ebi.ac.uk/chembl/, accessed on 20 June 2025). Several bioactivity metrics were present in the initial dataset, like, the half-maximal effective concentration (EC_50_), minimum inhibitory concentration (MIC), percentage of biological activity, inhibition constant (Ki), percentage of inhibition, and half-maximal inhibitory concentration (IC_50_). Only IC_50_ (half-maximal inhibitory concentration) values are converted to nanomolar (nM) and were selected for further analysis. Later, compounds with missing values, duplicate entries, and molecules with incomplete or missing bioactivity data were eliminated. Then molecules were categorized based on their bioactivity IC_50_ values, those with IC_50_ below 1000 nM were categorized as actives, 1000–10,000 nM as intermediate and those above 10,000 nM as inactive.

For data preprocessing, we used Google Collab and installed the RDKit library, which was originally managed within an Anaconda environment to calculate Lipinski’s Rule of Five that provided a guideline for predicting the oral bioavailability of compounds based on physicochemical descriptors [44]. This rule, developed by Christopher Lipinski, is a useful guideline in drug discovery, as it describes molecular properties that affect essential pharmacokinetic characteristics like absorption, distribution, metabolism, and excretion (ADME) [45]. Using Lipinski’s criteria, we used python libraries such as Matplotlib version 3.2.2 and Seaborn version 0.11.2 to visualize and analyze the chemical space of hDHFR inhibitors, focusing on active and inactive compounds after removing the intermediate class. Also, to normalize the distribution of pIC_50_ values, we converted the IC_50_ values to a negative logarithmic scale (pIC_50_) by using the formula pIC_50_ = −log_10_ (IC_50_).

### 3.2. Descriptors Calculation and Feature Selection

The most common types of two-dimensional molecular fingerprints (FPs) include structural-key FPs, topological or path-based FPs, circular FPs, pharmacophore FPs, and neural network-based FPs [46]. In this study three different fingerprints were employed to convert SMILES representations into binary feature vectors, using PubChem fingerprints, Substructure fingerprints, and MACCS fingerprints since they are also among the most commonly used ones in the literature [47,48,49,50,51,52,53]. These chemical fingerprints serve as input features for machine learning, enabling efficient training and evaluation of subsequent models. All fingerprints were calculated using PaDELPy (version 0.1.12) library (https://github.com/ecrl/padelpy, accessed on 20 August 2025) from the PaDEL-Descriptor software version 2.21 suite. Each chemical fingerprint serves as an input feature for machine and deep learning models, enabling efficient training and testing for predictive models [54]. Feature selection is a crucial preprocessing step in predictive modeling, as it helps avoids overfitting and weak predictivity, enhance model performance [55], and improve computational efficiency by selecting only the most relevant features for the model [56]. Once molecular features were calculated, feature selection approach was applied using recursive feature elimination (RFE) to obtain a reduced subset. The RFE method is a machine learning technique that iteratively eliminates less important features based on model performance, aiming to identify the optimal subset that enhances predictive accuracy [33,57].

### 3.3. Data Splitting

After selecting the key molecular descriptors from the three types of structural fingerprints, each dataset was randomly split into training and test sets using 80:20 ratio, and these sets were then applied to build three ML-QSAR models, which correlate molecular features and pIC_50_ value using the regression approach for bioactivity prediction. In the training set, molecular descriptors were selected and the performance of the model was evaluated on the test set, also known as external set [58]. This is an important step in building good models that generalize well, do not overfit, keep the bias low and that learn to perform well on other data instead of memorizing the training data [59]. As a result, data splitting provides reliable performance assessments and offers valuable insights into how chemical structures relate to biological activity [60].

### 3.4. ML-QSAR Models Optimization and Training

Principal Component Analysis (PCA) with Mahalanobis distance was used to perform the applicability domain analysis for each ML-QSAR prediction model and to remove outliers. Three-dimensional and 2D plots were created based on the first three principal components (PCA1, PCA2, and PCA3). Compounds that showed unexpected or poorly fitting activity, or are outside the Applicability Domain (AD), were considered outliers [61]. The Applicability Domain (AD) refers to the chemical space defined by the training data used to build a predictive model [62]. Instead, a clearly defined AD is an essential component in computational modeling systems, and the Organization for Economic Co-operation and Development (OECD) has incorporated AD as a requirement for QSAR models [63]. Hyperparameters tuning was performed using GridSearchCV (Scikit-learn, version 0.23.1) with 5-fold cross-validation. This approach combined grid search with five-fold cross-validation to find the optimal hyperparameters to be used in this machine learning study on the training set and for the features selected by Recursive Feature Elimination (RFE). The cross-validation results are used to select the hyperparameters of the different algorithms, balancing bias and variance [64]. Grid Search is an exhaustive search algorithm for hyperparameter optimization in ML models. Hyperparameter optimization has been well-studied, and many search methods like grid search and random search are commonly utilized [65]. Each model was trained and tested on every hyperparameter combination, with the optimal configuration determined by the highest validation performance. The final tuned hyperparameters are summarized in Table 5.

Based on the identified significant molecular features and optimal hyperparameter, a random forest regression model was subsequently trained and evaluated on both training and testing subsets for each fingerprint type to develop three robust ML-QSAR models, including PubChem, Substructure and MACCS fingerprints, to relate the molecular features with pIC_50_ values.

Random forest (RF) is a supervised machine learning technique that, as individual predictors, utilizes an ensemble of decision trees and aggregates their predictions to reduce the tendency of individual trees to overfit the data and enhance overall model performance [66]. By training multiple trees on different data subsets, RF effectively minimize overfitting and improve predictive accuracy on previously unseen data [67]. It is also appropriate for dealing with unbalanced data and is not sensitive to uninformative variables and outliers [68]. Many studies have shown that RF is advantageous in many respects, including its resistance to overfitting, efficient learning, robustness to noise data, and the ability to evaluate the importance of variables effectively [69].

### 3.5. Validation and Interpretation of the ML-QSAR Models

Performance is generally measured in regression problems using a set of standard metrics, including mean absolute error (MAE), mean squared error (MSE), root mean squared error (RMSE), R-squared, and Q-squared score. These metrics offer valuable insights into both the accuracy of predictions and the model’s generalization capability to unseen data [31]. Scikit-learn package (version 0.23.1) was used for calculating various validation metrics like R-squared (R^2^), MAE, MSE and RMSE for all ML-QSAR prediction models as shown in Equations (1), (2), (3) and (4), respectively.

The R-squared (R^2^) score represent the proportion of variance in the dependent variable from the independent variables, it is ranging from 0 (no fit) to 1 (perfect fit) [70]. MAE indicates the mean absolute differences in predictions and observations [71]. MSE measures the average of the squared differences between the actual data feature and the predicted data point generated by the model [72]. RMSE is another metric that represents the square root of MSE, reflecting the average magnitude of the prediction errors [73].

(1) $$R^{2}=1-\frac{\sum_{i=1}^{n}{\left(y_{actual\left(i\right)}-y_{predicted\left(i\right)}\right)}^{2}}{\sum_{i=1}^{n}{\left(y_{actual\left(i\right)}-y_{meanactual}\right)}^{2}}$$

(2) $$MAE=\left(\frac{1}{n}\right)\sum_{i=1}^{n}\left|y_{actual\left(i\right)}-y_{predicted\left(i\right)}\right|$$

(3) $$MSE=\left(\frac{1}{n}\right)\sum_{i=1}^{n}{\left(y_{actual\left(i\right)}-y_{predicted\left(i\right)}\right)}^{2}$$

(4) $$RMSE=\sqrt{\left(\frac{1}{n}\right)\sum_{i=1}^{n}{\left(y_{actual\left(i\right)}-y_{predicted\left(i\right)}\right)}^{2}}$$

where n is the total number of data points (observations); $y_{actual\left(i\right)}$ and $y_{predicted\left(i\right)}$ are the ith observation of the actual and predicted data, respectively; and $y_{meanactual}$ the mean of the actual data.

We also used feature importance scores from the random forest regressor for further insight into the importance of the different descriptors in models’ predictions. Numerically, RF-FI plots refer to the importance of features in the response variable. Higher RF-FI scores indicate that descriptors contribute more to explaining the variance in the response variable, offering important information about the main factors influencing the model’s predictions [25]. To complement this, a correlation matrix was constructed using the top molecular descriptors identified from the RF-FI analysis, allowing us to assess the degree of correlation among the most influential features.

## 4. Conclusions

In this study, three machine learning-based QSAR prediction models utilizing various molecular features to identify key structural characteristics of hDHFR inhibitors have been developed. The dataset was curated from the CHEMBL database, and molecular descriptors were generated using PaDEL. Subsequently, the top 50 features were selected through Recursive Feature Elimination combined with random forest regression (RFE-RFR) and used to build the regression models. Our results show that our models have good prediction performances. All models were then analyzed by applying important feature methods to quantify the impact of key input features and better understand how the model makes its predictions. The most significant molecular features among ML-QSAR prediction models were identified using random forest feature importance plots. PubchemFP420, PubchemFP374, PubchemFP372, PubchemFP540, PubchemFP553, PubchemFP712, PubchemFP528, PubchemFP659, PubchemFP643, and PubchemFP702, were selected as best features for PubChem prediction model. SubFP32, SubFP1, SubFP182, SubFP287, SubFP84, SubFP18, SubFP16, SubFP100, SubFP169 and SubFP2, for substructure prediction model, and MACCSFP116, MACCSFP119, MACCSFP78, MACCSFP104, MACCSFP96, MACCSFP90, MACCSFP144, MACCSFP154, MACCSFP97, and MACCSFP110, for MACCS prediction model. To benefit the scientific community, the developed ML-QSAR models were further deployed through a Python web application, (https://hdhfr-prediction.streamlit.app/, accessed on 10 September 2025) using the Streamlit library. Despite their strong overall performance, it is crucial to recognize the model’s limitation in accurately predicting the peak potency of ultra-active compounds. This reflects the inherent difficulty of modeling extreme values and highlights key opportunities for methodological improvement. Future work, as outlined above, will focus on addressing this specific shortcoming by exploring alternative machine learning algorithms, implementing more rigorous feature selection and applicability domain analysis, and incorporating additional data sources to enhance predictive accuracy, particularly for high-affinity binders. Increasing model interpretability will also remain a priority for gaining deeper insights into the structural determinants of potent inhibition.

## Figures and Tables

**Table 1:** Comparison of molecular descriptors between active and inactive compounds (mean ± SD) with statistical significance from Student’s *t*-test.

Descriptor	Active (Mean ± SD)	Inactive (Mean ± SD)	*t*-Value	*p*-Value	Significance
MW (Da)	391.24 ± 91.17	371.84 ± 86.78	2.966	0.003	**
Log *p*	2.53 ± 1.40	2.63 ± 1.40	−0.991	0.322	ns
HBA	6.98 ± 1.77	6.69 ± 1.91	2.176	0.030	*
HBD	3.10 ± 1.72	2.72 ± 1.27	3.393	0.001	***

*** *p* < 0.001 (Extremely significant); ** *p* < 0.01 (Very significant); * *p* < 0.05 (Significant); ns *p* ≥ 0.05 (Not significant).

**Table 2:** Performance evaluation metrics of all the constructed ML-QSAR models.

	PubChem Fingerprints		Substructure Fingerprints		MACCS Fingerprints	
Evaluation Metrics	Train (774)	Test (193)	Train (796)	Test (199)	Train (769)	Test (181)
R-squared (R^2^)	0.9934	0.9591	0.9849	0.9381	0.9924	0.9381
MAE	0.0593	0.1250	0.0865	0.1381	0.0642	0.1397
MSE	0.0070	0.0342	0.0159	0.0484	0.0085	0.0446
RMSE	0.0837	0.1848	0.1261	0.2199	0.0919	0.2111

The number of molecules used for training and testing are listed in brackets for the different fingerprints.

**Table 3:** Description of the top 10 molecular features of all ML-QSAR models.

Fingerprints	Description	Fingerprints	Description	Fingerprints	Description
PubchemFP 420	C=O	SubFP32	Tertiary arom amine	MACCSFP116	Aromatic ring with a pyrazole group
PubchemFP 374	C(~H)(~H)(~H)	SubFP1	Primary carbon	MACCSFP119	Aromatic ring with a chloro group
PubchemFP 372	C(~H)(:C)(:N)	SubFP182	Hetero O	MACCSFP78	Aromatic ring with a chlorine group
PubchemFP 540	C-N-C-[#1]	SubFP287	Conjugated double bond	MACCSFP104	Aromatic ring with a alkyl group
PubchemFP 553	O=C-C=C	SubFP84	Carboxylic acid	MACCSFP96	Aromatic ring with a nitrile group
PubchemFP 712	C-C(C)-C(C)-C	SubFP18	Alkylarylether	MACCSFP90	Aromatic ring with a nitro group
PubchemFP 528	[#1]-N-C-[#1]	SubFP16	Dialkylether	MACCSFP144	Aromatic ring with a chloro group
PubchemFP 659	O-C-C-N-C	SubFP100	Secondary Amide	MACCSFP154	Aromatic ring with a alkyl group
PubchemFP 643	[#1]-C-C-N-[#1]	SubFP169	Phenol	MACCSFP97	Aromatic ring with a sulfonic acid group
PubchemFP 702	O-C-C-C-C-C-N-C	SubFP2	Secondary carbon	MACCSFP110	Aromatic ring with an epoxide group

**Table 4:** Experimental vs. Predicted pIC50 Values of hDHFR Inhibitors Using Three Fingerprint Models.

Compound	Experimental pIC50	Predicted PubChem	Predicted Substructure	Predicted MACCS
Methotrexate	9.08	7.65	7.17	7.37
CHEMBL83644	8.88	8.32	7.09	7.38
CHEMBL160699	8.87	7.14	7.09	8.26
CHEMBL18925	7.72	7.36	7.64	7.50

**Table 5:** Hyperparameters Grid for Model Tuning.

Hyperparameter	Selected Values
n_estimators max_features max_depth	10, 50, 100, 500, 1000 auto, sqrt, log2 5, 10, 20, 30, 50

## Data Availability

The source code and web application developed in this study are publicly available in our GitHub repository at: https://github.com/IbMaat/hDHFR accessed on 20 August 2025. The Streamlit web application can be accessed at: https://hdhfr-prediction.streamlit.app/ accessed on 10 September 2025. The raw bioactivity data used for model training were obtained from the ChEMBL database (https://www.ebi.ac.uk/chembl/ accessed on 20 June 2025) and processed as described in the Section 2. Processed descriptor matrices and model training files are available from the corresponding author upon reasonable request.

## Abbreviations

The following abbreviations are used in this manuscript:

AD	Applicability Domain
ChEMBL	European Molecular Biology Laboratory Chemical Database
DNA	Deoxyribonucleic Acid
FDA	U.S. Food and Drug Administration
FPs	Fingerprints
hDHFR	Human dihydrofolate reductase
IC50	Half-Maximal Inhibitory Concentration
MACCSFP	MACCS Fingerprint
MAE	Mean Absolute Error
MSE	Mean Squared Error
ML	Machine Learning
ML-QSAR	Machine Learning-based Quantitative Structure–Activity Relationship
PCA	Principal Component Analysis
PubChemFP	PubChem Fingerprints
pIC50	Negative Logarithmic Scale of IC50
R^2^	Coefficient of Determination (R-squared)
RFE	Recursive Feature Elimination
RFR	Random Forest Regression
RMSE	Root Mean Square Error
RNA	Ribonucleic Acid
THF	Tetrahydrofolate
SMILES	Simplified Molecular Input Line Entry System
SubFP	Substructure Fingerprints
RF-FI	Random Forest Feature Importance
